# Effect of Driving Pressure-Oriented Ventilation on Patients Undergoing One-Lung Ventilation During Thoracic Surgery: A Systematic Review and Meta-Analysis

**DOI:** 10.3389/fsurg.2022.914984

**Published:** 2022-05-27

**Authors:** Xuan Li, Wenqiang Xue, Qinyu Zhang, Yuyang Zhu, Yu Fang, Jie Huang

**Affiliations:** Department of Anesthesiology, The First Affiliated Hospital of Kunming Medical University, KunMing, China

**Keywords:** driving pressure, lung-protective ventilation, one-lung ventilation (OLV), oxygenation, postoperative pulmonary complications (PPCs)

## Abstract

**Background:**

Hypoxemia and fluctuations in respiratory mechanics parameters are common during one-lung ventilation (OLV) in thoracic surgery. Additionally, the incidence of postoperative pulmonary complications (PPC_S_) in thoracic surgery is higher than that in other surgeries. Previous studies have demonstrated that driving pressure-oriented ventilation can reduce both mortality in patients with acute respiratory distress syndrome (ARDS) and the incidence of PPC_S_ in patients undergoing general anesthesia. Our aim was to determine whether driving pressure-oriented ventilation improves intraoperative physiology and outcomes in patients undergoing thoracic surgery.

**Methods:**

We searched MEDLINE via PubMed, Embase, Cochrane, Web of Science, and ClinicalTrials.gov and performed a meta-analysis to compare the effects of driving pressure-oriented ventilation with other ventilation strategies on patients undergoing OLV. The primary outcome was the PaO_2_/FiO_2_ ratio (P/F ratio) during OLV. The secondary outcomes were the incidence of PPC_S_ during follow-up, compliance of the respiratory system during OLV, and mean arterial pressure during OLV.

**Results:**

This review included seven studies, with a total of 640 patients. The PaO_2_/FiO_2_ ratio was higher during OLV in the driving pressure-oriented ventilation group (mean difference [MD]: 44.96; 95% confidence interval [CI], 24.22–65.70.32; *I*^2^: 58%; *P* < 0.0001). The incidence of PPC_S_ was lower (OR: 0.58; 95% CI, 0.34–0.99; *I*^2^: 0%; *P* = 0.04) and the compliance of the respiratory system was higher (MD: 6.15; 95% CI, 3.97–8.32; *I*^2^: 57%; *P* < 0.00001) in the driving pressure-oriented group during OLV. We did not find a significant difference in the mean arterial pressure between the two groups.

**Conclusion:**

Driving pressure-oriented ventilation during OLV in patients undergoing thoracic surgery was associated with better perioperative oxygenation, fewer PPC_S_, and improved compliance of the respiratory system.

**Systematic Review Registration:**

PROSPERO, identifier: CRD42021297063.

## Introduction

One-lung ventilation (OLV) has been widely used in thoracic surgery to isolate and protect the lungs (1). However, in the process of OLV, the lung on the non-operated side is still perfused, which causes intrapulmonary shunting (2). This, along with the lateral decubitus positioning of the patient and the intrathoracic pressure imbalance, causes an impairment in ventilation-perfusion matching, a decrease in the compliance of the respiratory system (C_RS_), and hemodynamic fluctuations (3), all of which may impair arterial oxygenation. Hypoxemia during OLV has always been the most difficult problem for anesthesiologists to overcome (4). These perioperative changes in physiological parameters, combined with mechanical ventilation-induced lung injury, result in a higher incidence of postoperative pulmonary complications (PPC_S_) in patients undergoing thoracic surgery than in those undergoing other types of surgery (5, 6). Therefore, the choice of intraoperative ventilation strategy is crucial.

The concept of driving pressure was first proposed in regards to patients with ARDS (defined according to the Berlin definition) (7). To minimize ventilator-induced lung injury, most studies scaled tidal volume (V_T_) by predicted body weight, making the V_T_ facility more closely matched to the patient’s lung size (8, 9). However, in patients with ARDS, the C_RS_ is lower, which significantly reduces the proportion of the lungs that can be ventilated (8, 9). Therefore, we hypothesized that, compared with using V_T_ alone, attributing the V_T_ to the C_RS_ and using the quotient of the two as a predictor of functional lung size could be used to better predict the prognosis of patients with ARDS (7). Therefore, the driving pressure, which is defined as V_T_/C_RS_, can simply be calculated as the difference between the plateau pressure and positive end-expiratory pressure (PEEP) (7). Recently, several retrospective studies and systematic reviews have shown that the driving pressure is positively associated with the incidence of ventilator-induced lung injury, with low driving pressure shown to reduce mortality in patients with ARDS (7, 10, 11). Therefore, the relationship between driving pressure and PPC_S_ may be more important than any other parameter (7, 11). Similarly, patients undergoing thoracic surgery with OLV tend to have a higher incidence of intraoperative hypoxemia and worse prognosis than patients with double-lung ventilation (DLV) because only the non-operated lung is ventilated. We hypothesized that such driving pressure-oriented ventilation, which minimizes the driving pressure during mechanical ventilation, would improve intraoperative physiological function and prognosis in patients with OLV.

Previous meta-analyses have demonstrated that a low driving pressure is associated with a lower incidence of PPC_S_ in patients with DLV (11), and recent randomized controlled trials (RCTs) have demonstrated that driving pressure-oriented ventilation improves outcomes in patients with OLV (1). To date, no meta-analysis has demonstrated the applicability of such ventilation strategies in patients with OLV. For this study, RCTs on driving pressure-oriented ventilation in thoracic surgery were reviewed and a meta-analysis was performed to investigate whether driving pressure-oriented ventilation improves intraoperative physiological function and prognosis in patients undergoing OLV.

## Method

### Search Strategy

Ethical approval and patient consent were not required as this was a systematic review and meta-analysis of previously published studies. The study has been registered in PROSPERO. (http://www.crd.york.ac.uk/prospero), registration number: CRD42021297063.This systematic review and meta-analysis was conducted following the PRISMA guidelines. (Supplementary Table S1). (12) The following databases were searched for relevant research in back-to-back experiments by two authors (XL and WX) : MEDLINE via PubMed, Embase, Cochrane, Web of Science, and ClinicalTrials.gov from the first record to December 1, 2021. The search formula is as follows: (“one-lung ventilation”[Title/Abstract] OR “one lung ventilation”[Title/Abstract] OR “single lung ventilation”[Title/Abstract] OR “OLV”[Title/Abstract] OR “thoracic surgery”[Title/Abstract]) AND (“driving pressure”[Title/Abstract]). Disagreements were resolved by discussion with another author (YF).

### Inclusion and Exclusion Criteria

The inclusion criteria for the study followed the following strategies: (1) Design: Results from our search strategy were limited to randomized controlled trials (RCTs) and human experiments. (2) Age and surgery: Adult (age >18 years) patients undergoing one-lung ventilation for thoracic surgery. (3) Interventions: RCTs using driving pressure-oriented ventilation or RCTs using other ventilation strategies but recording driving pressure during the study. (4) Eligible studies must report oxygenation index or partial pressure of oxygen and must report at least one of the following outcomes: compliance of the respiratory system, mean arterial pressure or incidence of postoperative pulmonary complications. The exclusion criteria for the study followed the following strategies: (1): case reports. (2): observational studies. (3): reviews. (4): Using a driving pressure-oriented ventilation strategy but not thoracic surgery. Two researchers screened all studies after excluding duplicate studies and screened references of included studies for additional relevant studies.

### Outcome Measures

Primary outcomes: The PaO_2_/FiO_2_ ratio of patients during one-lung ventilation.

Secondary outcomes: (1) Incidence of postoperative pulmonary complications during follow-up. Postoperative pulmonary complications were assessed using the Melbourne Group Scale: chest x-ray findings of atelectasis or consolidation; raised white cell count [greater than 11.2 × 10^6^/mL] or administration of respiratory antibiotics postoperatively, in addition to prophylactic antibiotics; temperature greater than 38°C; signs of infection on sputum microbiology; purulent sputum different from preoperative status; oxygen saturation less than 90% on room air; physician diagnosis of pneumonia; and prolonged intensive care unit stay [longer stay than 1 and 2 days for lung and esophagus surgery, respectively] or readmission to the intensive care unit. (13) (2) compliance of the respiratory system (C_RS_) during one-lung ventilation. (3) Mean arterial pressure (MAP) during one-lung ventilation.

#### Subgroup Analysis

For continuous variables: P/F ratio, C_RS_, and MAP, due to the different time points of measurement, three experiments recorded the time points at which they were measured, and we performed subgroup analysis for the three outcomes.

### Data Extraction and Risk of Bias Assessment

Two authors (XL and WX) screened the titles and abstracts of initial search results, extracted data, and independently assessed the risk of bias. Get more information by directly asking the corresponding author in the relevant article if needed. Each randomized trial was assessed using the Cochrane Library’s RCT Risk of Bias tool, taking into account the following possible sources of bias. The methodological quality of the trial: random sequence generation; allocation concealment; blinding of participants and raters of lost-to-follow outcomes; incomplete outcome data; selective outcome reporting and other biases. And classify it as “low”, “high” or “unclear” risk. (Figure 2). Disagreements were resolved by discussion with another author (YF).

### Statistical Analysis

We performed meta-analyses using Review Manager software (RevMan version 5.4). The coefficient *I*^2^ was calculated to assess heterogeneity, which was defined as low (25%–49%), medium (50%–74%), and high (>75%) levels. A random-effects model was used for all analyses due to clinical methodological heterogeneity and other potential heterogeneities. Whenever there was significant heterogeneity, we performed a meta-analysis by omitting one study in turn to find potential sources of heterogeneity. Publication bias was due to the limited number of included studies (<10) and was not assessed. We calculated odds ratios (OR) using 95% CI for dichotomous variables and mean differences (MD) for continuous variables. When reporting continuous results as medians and interquartile ranges in some studies, we used the method described by McGrath et al. (14) to estimate the mean and standard deviation for data pooling for continuous variables. *P* < 0.05 was considered The difference is statistically significant.

## Results

### Selection of Studies

**Figure 1:**
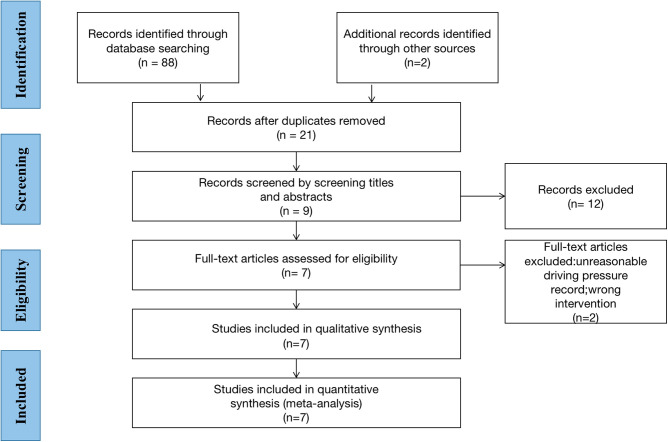
Flow diagram of the study selection.

**Figure 2:**
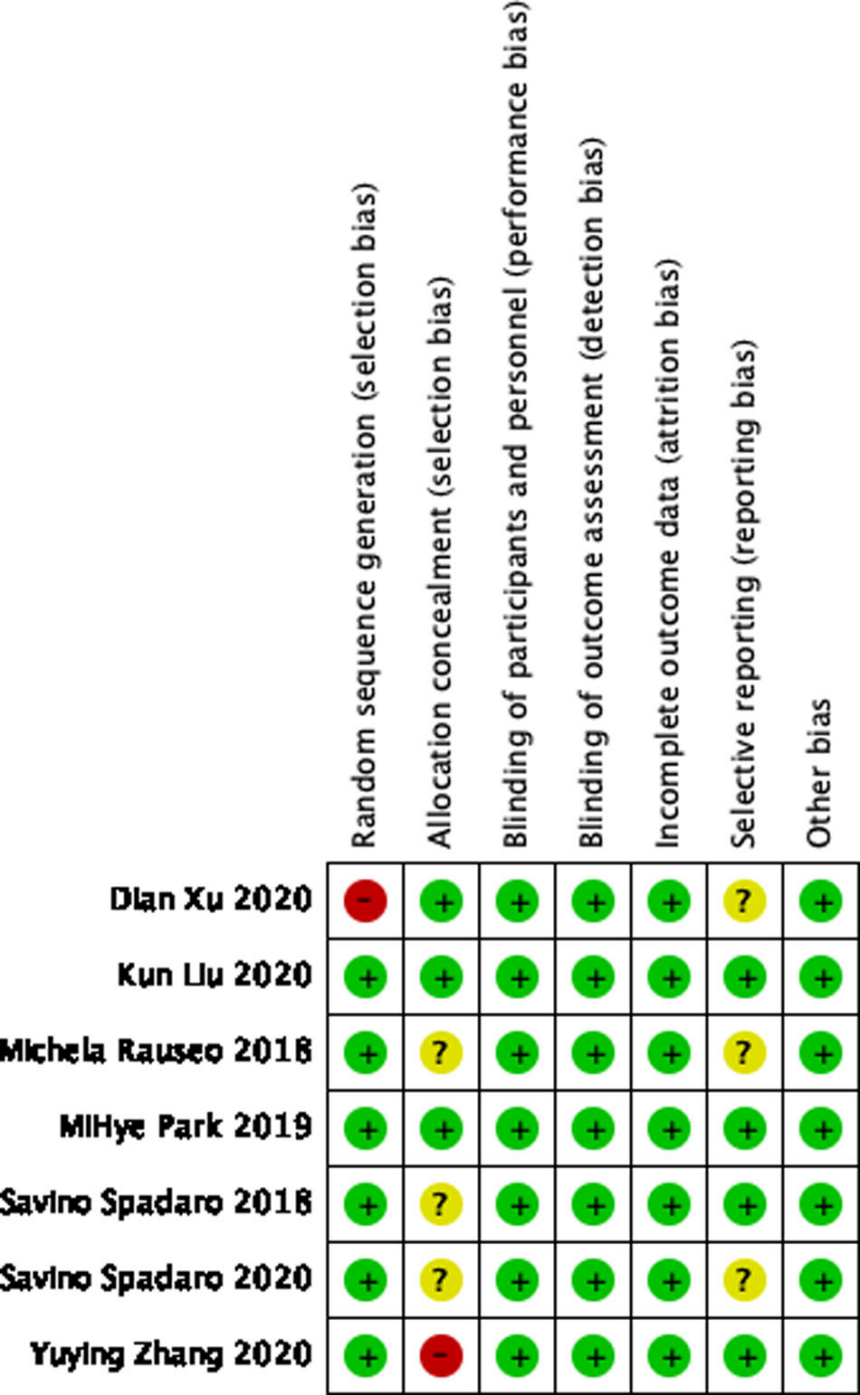
Risk of bias assessment of the included studies.

Following the search strategy described above, we obtained 88 relevant articles from our initial search results and two relevant articles from manually reviewing the reference lists of the studies (Supplementary Table S2). Two authors (XL and WX) screened nine studies by reading the titles and abstracts and removing duplicate studies, non-randomized controlled trials, experimental reports, and reviews. After carefully reading the full text of the nine studies, two were excluded and only seven studies were included for full-text evaluation (1, 15–20). A total of 640 patients were included in these seven studies (Figure 1). All patients were adults with an American Society of Anesthesiologist physical status I-III undergoing OLV for thoracic surgery. The Cochrane Collaboration risk of bias tool (Figure 2) indicated that the risk of bias was low for most of the trials.

Table 1 shows the basic characteristics of the included studies. Since the RCTs of individualized PEEP we included all recorded intraoperative driving pressure, and the driving pressure of the experimental group was lower than that control group and this difference was statistically significant, we called “driving pressure-oriented”, that is, to decrease driving pressure during mechanical ventilation, so our study defines the experimental group of these studies as the driving pressure-oriented group. The reasons and details for our inclusion of these studies are provided in Supplementary Table S3.

**Table 1:** Characteristics of the included studies.

Reference	Country	NOS score	Population(n)	Surgery	Experimental Group	Control Group	Outcomes
Park et al. (1)	Korea	9	Control: *n* = 147 Driving pressure-oriented: *n* = 145	Elective thoracic surgery	Driving pressure-oriented by PEEP titration	Protective ventilation:PEEP = 5 cmH_2_O	PPC_S_. Intraoperative arterial blood gas analysis
Spadaro et al (17)	Italy	8	Control: *n* = 41 Driving pressure-oriented: *n* = 41	elective lobectomy, lung resection	PEEP = 10 cmH_2_O	PEEP = 0 cmH_2_O	Shunt fraction Respiratory mechanics Oxygenation
Liu et al (15)	China	8	Control: *n* = 50 Driving pressure-oriented: *n* = 50	Pneumonectomy, wedge resection, lobectomy, wedge + lobectomy	Driving pressure-oriented by EIT	PEEP = 5 cmH_2_O	Oxygenation Respiratory mechanics PPC_S_
Rauseo et al (18)	Italy	6	Control: *n* = 13 Driving pressure-oriented: *n* = 13	Elective lung lobectomy or resection	Driving pressure-oriented by open lung approach	*V_T_*: 6–8 mL/PBW PEEP = 0 cmH_2_0	Oxygenation Respiratory mechanics hemodynamics
Spadaro et al. (19)	Italy	6	Control: *n* = 13 Driving pressure-oriented: *n* = 13	lobectomy or wedge resection	Driving pressure-oriented by stepwise decrease PEEP from 16 cmH_2_O after a lung recruiting manoeuvre	PEEP = 0 cmH_2_0	Respiratory mechanics ventilation/perfusion Mismatch Oxygenation
Xu et al. (20)	China	7	Control: *n* = 15 Driving pressure-oriented: *n* = 15	Elective pulmonary resection or esophagectomy	Driving pressure-oriented by titrate PEEP to achieve maximum dynamic compliance	PEEP = 0 cmH_2_0	Oxygenation Respiratory mechanics hemodynamics
Zhang et al. (16)	China	7	Control: *n* = 29 Driving pressure-oriented: *n* = 29	Elective thoracoscopic lobectomy	Driving pressure-oriented by PEEP decremental trial	PEEP = 5 cmH_2_O	Hemodynamics Oxygenation PPC_S_

### Primary Outcomes

**Figure 3:**
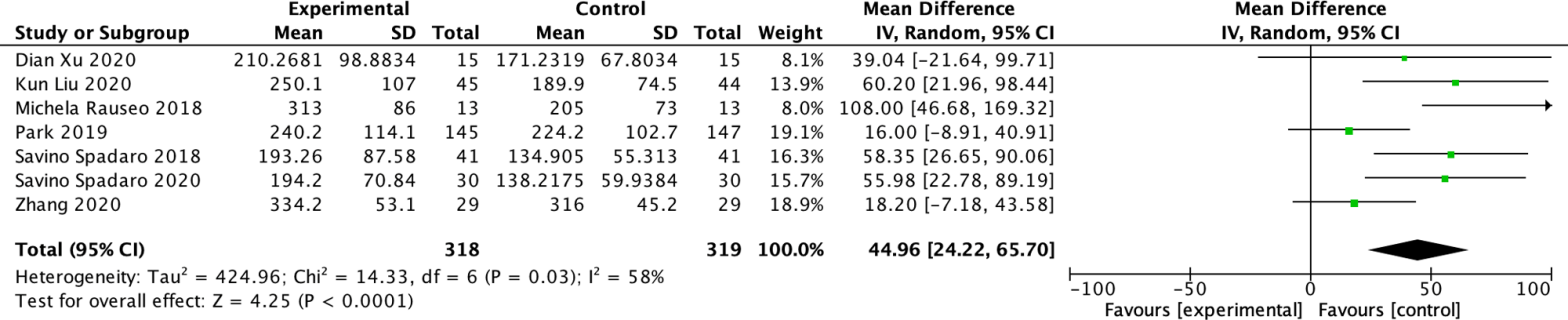
Forest plot of the PaO_2_/FiO_2_ ratio during one-lung ventilation.

Among the included studies, five reported PaO_2_/FiO_2_ ratio (15, 17–20) and two studies reported the PaO_2_ and FiO_2_ (FiO_2 _= 100% for both studies). (1, 16) In one study, the PaO_2_/FiO_2_ ratio was measured after 15 min of OLV (1), in another study, it was measured after 20 min of OLV (18), and in three studies, it was measured after 30 min of OLV (15, 16, 20). Two studies did not mention the specific time of measurement (17, 19). The PaO_2_/FiO_2_ ratio decreased in both groups during OLV compared with DLV in all the included studies. The PaO_2_/FiO_2_ ratio of the driving pressure-oriented group was higher during OLV (MD: 44.96; 95% CI, 24.22–65.70; *I*^2^: 58%; *P* < 0.0001) (Figure 3).

### Secondary Outcomes

**Figure 4:**
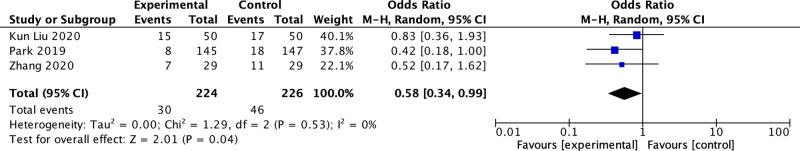
Summary data forest plot of the number of patients with postoperative pulmonary complications.

Three studies reported PPC_S_ (1, 21, 22). All reports followed up on PPC_S_ during the postoperative hospital stay, and the total number of patients with PPC_S_ during postoperative follow-up was included in our analysis. A random effects model was applied, and the incidence of PPC_S_ was found to be 30/224 (13.4%) in the driving pressure-oriented group and 46/226 (20.4%) in the control group. The incidence of PPC_S_ in the driving pressure-oriented group was lower than that in the control group (OR: 0.58; 95% CI, 0.34–0.99; *I*^2^: 0%; *P* = 0.04) (Figure 4).

**Figure 5:**
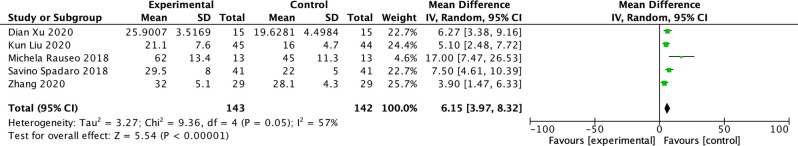
Forest plot of respiratory system compliance during one-lung ventilation.

**Figure 6:**
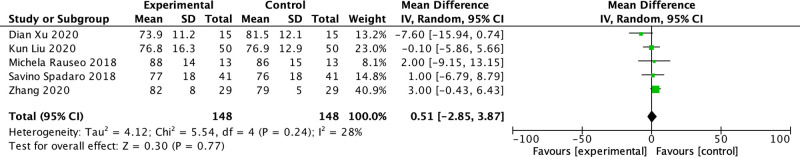
Forest plot of mean arterial pressure during one-lung ventilation.

The C_RS_ during OLV was reported in five studies (15–18, 20). The C_RS_ was found to be higher in the driving pressure-oriented group than in the control group (MD: 6.15; 95% CI, 3.97–8.32; *I*^2^: 57%; *P* < 0.00001) (Figure 5). Five studies reported mean arterial pressure (MAP) during OLV (15–18, 20). We did not find a significant difference in the MAP between the two groups during OLV (MD: 0.51; 95% CI, −2.85–3.87; *I*^2^: 28%; *P* = 0.77) (Figure 6).

### Subgroup Analysis

Three studies recorded the PaO_2_/FiO_2_ ratio at 0.5 h of OLV, (15, 16, 20) and two studies recorded the PaO_2_/FiO_2_ ratio at 1 h of OLV. (15, 20) PaO_2_/FiO_2_ ratio was higher in the driving pressure-oriented group at 0.5 h.(MD: 35.64; 95% CI, 7.38–63.90; *I*^2^: 39%; *P* = 0.01) (Supplementary Figure S1). There was no significant difference compared with the control group at 1 h. (Supplementary Figure S2)

At 0.5 h and 1 h of OLV, the C_RS_ of the driving pressure-oriented group was lower than the control group (MD: 4.63; 95% CI, 2.71–6.54; *I*^2^: 62%; *P* < 0.00001), (MD: 5.63; 95% CI, 3.69–7.57; *I*^2^: 0%; *P* < 0.00001) (Supplementary Figures S3, S4).

At 0.5 h and 1 h of OLV, we didn’t find a significant difference in MAP between the driving pressure-oriented group and the control group (Supplementary Figures S5, S6).

### Sensitivity Analysis and Publication Bias

We detected a moderate degree of heterogeneity regarding our primary outcome and performed a sensitivity analysis to explore potential reasons for this. The heterogeneity was reduced to 55%, and the mean difference (MD) changed from 44.96 to 51.50 after excluding the study conducted by Zhang et al. (16) The heterogeneity was reduced to 50%, and the MD changed from 44.96 to 51.42 after excluding the study conducted by Park et al. (1) The heterogeneity was reduced to 0%, and the MD changed from 44.96 to 60.62 after excluding both studies (1, 16).

**Figure 7:**
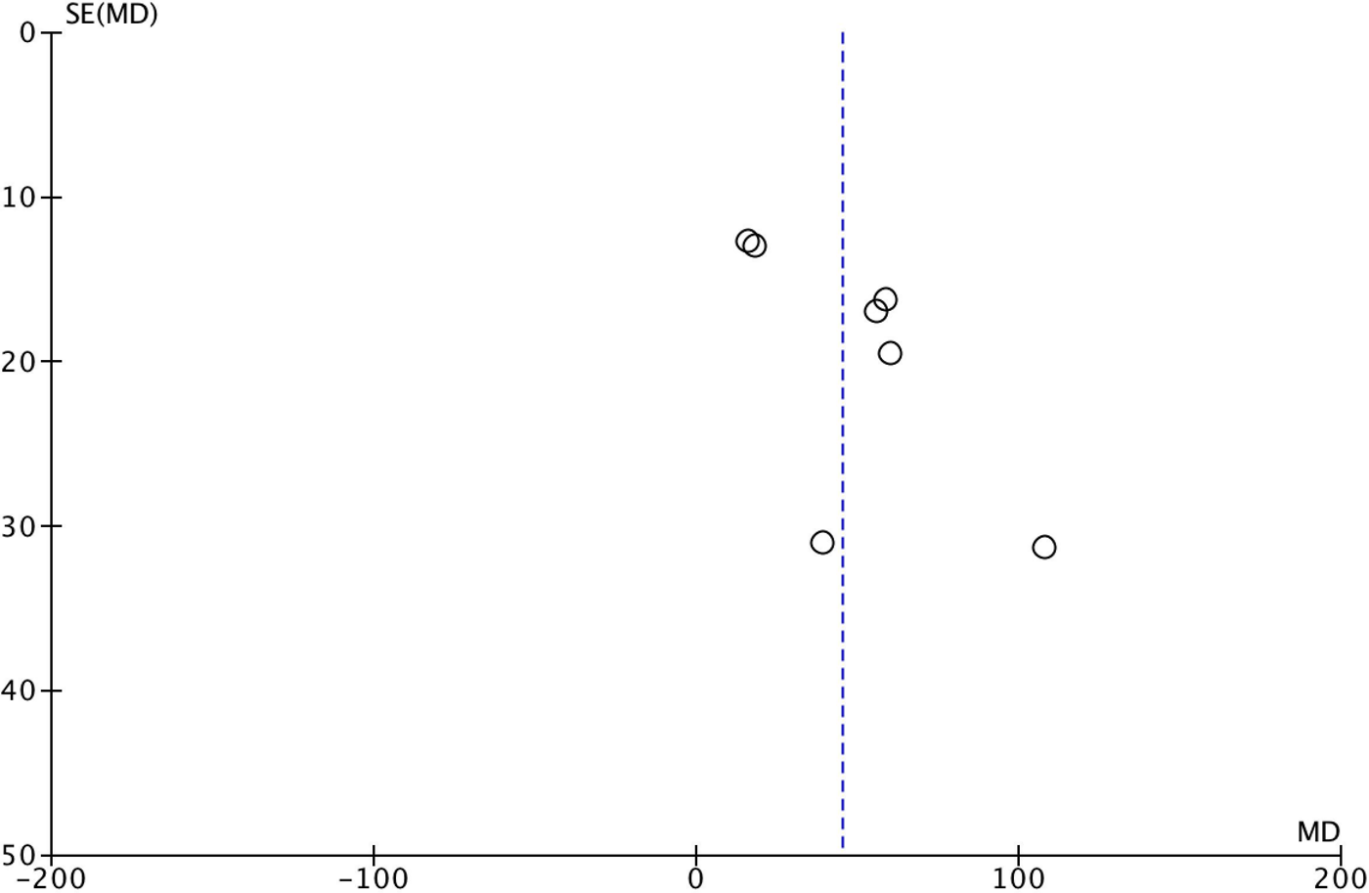
Funnel plot for meta- of the PaO_2_/FiO_2_ ratio.

Funnel plots were used to evaluate the publication bias of the included studies. No evidence of publication bias for the primary outcome was suggested by visual inspection of the funnel plots. No significant publication bias was observed (Figure 7).

## Discussion

In this meta-analysis, we investigated and compared the effects of driving pressure-oriented ventilation with those of other ventilation strategies on intraoperative oxygenation, the C_RS_, MAP, and PPC_S_ in patients undergoing OLV. Our main finding was that driving pressure-oriented ventilation improved the intraoperative PaO_2_/FiO_2_ ratio (MD: 44.96; 95% CI, 24.22–65.70; *I*^2^: 58%; *P* < 0.0001), reduced the incidence of PPC_S_ (OR: 0.58; 95% CI, 0.34–0.99; *I*^2^: 0%; *P* = 0.04), improved the C_RS_ during OLV (MD: 6.15; 95% CI, 3.97–8.32; *I*^2^: 57%; *P* < 0.00001), and did not significantly alter the MAP during OLV (MD: 0.51; 95% CI, −2.85–3.87; *I*^2^: 28%; *P* = 0.77).

The vast majority of patients receiving general anesthesia develop atelectasis (23), which impairs intraoperative oxygenation. In patients undergoing thoracic surgery, OLV is prone to intrapulmonary shunting, imbalance in ventilation-perfusion matching, and the proportion of lung that can be ventilated is significantly reduced. These factors ultimately lead to a substantially increased incidence of hypoxemia with OLV, so the choice of ventilation strategy for thoracic surgery is crucial. In recent years, lung-protective ventilation (LPV), which aims to use low V_T_ and PEEP with the recruitment maneuver to prevent PPCs. (21, 22, 24, 25) However, low *V*_*T*_ does not appear to be associated with lower PPC_S_ (26–29). Moreover, individual characteristics, such as chest wall size and shape, abdominal contents, lung weight, and pleural pressure, vary from person to person, so a fixed PEEP may not be appropriate for everyone (30). Therefore, the definition of protective ventilation in thoracic surgery is still unclear. As we mentioned above, driving pressure can set the optimal *V*_*T*_ and PEEP. (7). Our results show that patients’ intraoperative PaO_2_/FiO_2_ ratio and C_RS_ with driving pressure-oriented ventilation were higher compared with other ventilation modalities. The results of the subgroup analysis showed that the PaO_2_/FiO_2_ ratio of the driving pressure group was still higher than that of the control group after 0.5 h of OLV. However, at 1 h of OLV, there was no significant difference in the PaO_2_/FiO_2_ ratio between the two groups. We speculate that this may be explained by the fact that the hypoxic pulmonary vasoconstriction response in the patients in the two groups took effect after 1 h of OLV, which reduces shunting and improves oxygenation, so in the study of Xu et al.(20). There was little difference in the PaO_2_/FiO_2_ ratio between the two groups (25). The subgroup analysis of the C_RS_ showed that the driving pressure group had a higher C_RS_ at both time points (0.5 h and 1 h), indicating that the effect of driving pressure on intraoperative respiratory mechanics was persistent over time. Therefore, driving pressure-oriented ventilation has a certain guiding effect on the setting of *V*_*T*_ and PEEP during ventilation and may become the target of a new protective ventilation strategy (31).

Inappropriate ventilation can exacerbate ventilator-induced lung injury and cause the release of a large number of inflammatory cytokines from lung endothelium and alveoli, which eventually leads to PPC_S_ (32, 33). The occurrence of PPC_S_ can delay patient recovery, increase the hospital length of stay and cost of hospitalization, and even lead to death. For many years, the use of LPV in thoracic surgery has been increasingly promoted to reduce the incidence of PPC_S_ (21, 22, 24); however, changes in the *V*_*T*_ and PEEP were not found to be associated with PPC_S_, or were found to be associated with PPC_S_ only when they caused changes in the driving pressure (7). Our findings suggest that driving pressure-oriented ventilation reduces the incidence of PPC_S_ in patients undergoing thoracic surgery, which is consistent with previous findings (1). Despite some heterogeneity in the type of surgery, the results of previous studies have demonstrated that driving pressure-oriented ventilation can reduce the incidence of PPC_S_ and improve patient prognosis (1, 34). Since driving pressure is a new concept, this strategy is more widely used in patients with ARDS, and the number of RCTs that have focused on this aspect of thoracic surgery is limited. Although there may be some limitations in terms of predicting the incidence of PPCs, the results did not suggest high heterogeneity. Our findings may provide some guidance in decision-making regarding intraoperative ventilation strategies for thoracic surgeries.

Since the operated lung is collapsed during OLV, the pleural pressure will be unbalanced, which may have a certain impact on hemodynamics during ventilation. However, our MAP results showed no significant differences in the MAP during OLV between the two groups. Subgroup analyses also showed no significant differences between the groups, indicating that the change in driving pressure may only have an effect on lung compliance or may only affect the lung itself, while its effect on the pleural pressure may be comparable to other modes of ventilation. However, only a few studies were included in our analysis, especially in the subgroup analysis. In addition, differences in the amount of intraoperative fluid infusion, the use of vasoactive drugs, and the method of anesthesia used in the included studies will affect hemodynamic fluctuations; therefore, our findings should be interpreted with caution. More RCTs are needed to compare the effects of driving pressure on hemodynamics.

Based on the results of sensitivity analysis. We carefully analyzed the differences between the two studies (1, 16) and the other studies and concluded that the high heterogeneity may be due to the following: (1) the intraoperative blood gas analyses were conducted at different time points; (2) the number of patients included in the study by Park et al. was larger than that in the other studies. Although they were all patients with OLV, there may have been some differences in the baseline characteristics of the patients from other studies; (3) a protective ventilation strategy was adopted for the control group in Park et al.’s study (*V*_*T*_: 6–8 mL/kg IBW, PEEP = 5 cmH_2_O, combined with a certain amount of lung recruitment maneuvers), while in the other included studies, the control group basically adopted conventional ventilation strategies and protective ventilation strategies were not exclusively used; and 4) Zhang et al.’s study was designed to titrate PEEP to the best C_RS_ and was not strictly driving pressure-oriented because the driving pressure of the experimental group was statistically significantly lower than that of the control group (*P* < 0.01). We thus regarded their experimental group as the driving pressure-oriented group. This difference in the ventilation mode may also help explain this heterogeneity.

Our study has some limitations. First, Our study included only seven RCTs with a total of 640 patients. It is difficult to draw Critical clinically significant conclusions from the small sample size. Second, not all of the trials included in the meta-analysis strictly titrated intraoperative ventilation parameters to the lowest driving pressure; rather, the driving pressure in the experimental group was statistically significantly lower than that in the control group. These experiments also used different ventilation settings, which may have had an impact on the results and degree of heterogeneity. Third, We initially decided to include RCTs and cohort studies on the PROSPERO protocol. However, the final search showed no relevant cohort studies, so we finally decided to include only RCTs. Furthermore, owing to the limited number of trials that reported the PPC_S_ and met our inclusion criteria, we could only assess the patients’ intraoperative PaO_2_/FiO_2_ ratio as the primary outcome. So there were several deviations from our original PROSPERO protocol (title, outcomes, and inclusion criteria). Moreover, the intraoperative blood gas analysis was performed at different times for each experiment. Fourth, since only two studies recorded intraoperative shunt fraction, we were not able to assess shunt fraction even though this may have reflected intraoperative oxygenation better than the C_RS_. Finally, PPC_S_ included postoperative lung injury, atelectasis, pulmonary infection, and barotrauma, among others, but only two studies provided subgroup data, so we did not perform a subgroup analysis of the PPC_S_. There are some more critical clinical outcomes - the ICU length of stay, hospital length of stay, and mortality. However, only one of our included studies recorded hospital length of stay (16), so we were unable to assess these more valuable clinical outcomes. For the continuous variables, since we considered that the results would be different depending on the time point, we performed a subgroup analysis. However, only three studies clearly recorded the monitoring time points of the continuous variables, which was insufficient. Therefore, the aggregate results of the subgroup analysis should be interpreted with caution.

## Conclusion

In patients undergoing thoracic surgery, driving pressure-oriented ventilation during OLV improves intraoperative oxygenation, reduces the incidence of PPC_S_, and improves C_RS_. However, more RCTs are necessary to confirm these findings.

## Data Availability

The original contributions presented in the study are included in the article/Supplementary Material, further inquiries can be directed to the corresponding author/s.
