# Supplementary material for: Effect of Driving Pressure-Oriented Ventilation on Patients Undergoing One-Lung Ventilation During Thoracic Surgery: A Systematic Review and Meta-Analysis
Source: Front Surg. 2022 May 27;9:914984. doi: 10.3389/fsurg.2022.914984 (PMC9198650; doi:10.3389/fsurg.2022.914984)
Supplement: Supplementary file 1 [file Table_2_v1.docx]

Supplementary Table 2: Individual search strategies for all the databases and the number of search results.

| Database | Search Strategy | Result |
| --- | --- | --- |
| MEDLINE via PubMed | (((((one-lung ventilation[Title/Abstract]) OR (one lung ventilation[Title/Abstract])) OR (single lung ventilation[Title/Abstract])) OR (OLV[Title/Abstract])) OR (thoracic surgery[Title/Abstract])) AND (driving pressure[Title/Abstract]) | 18 |
| Cochrane | #1: ("one lung ventilation"):ti,ab,kw OR ("one-lung ventilation"):ti,ab,kw OR ("single lung ventilation"):ti,ab,kw OR (OLV):ti,ab,kw OR ("thoracic surgery"):ti,ab,kw#2: ("driving pressure"):ti,ab,kw#3: #1 AND #2 | 18 |
| ClinicalTrials.gov | driving pressure \| Interventional Studies \| one-lung ventilation | 7 |
| Embase | #1: 'one lung ventilation':ab,ti OR 'one-lung ventilation':ab,ti OR 'single lung ventilation':ab,ti OR 'olv':ab,ti OR 'thoracic surgery':ab,ti  #2: 'driving pressure':ab,ti  #3: #1 AND #2 | 30 |
| Web of Science | **#1: TS=("one-lung ventilation" OR "one lung ventilation" OR "single lung ventilation" OR "OLV" OR "thoracic surgery")**  **#2: TS=("driving pressure")**  **#3: TS=(random* controlled trial OR random* OR placebo)**  **#4: #1 AND #2 AND #3** | 15 |
