# Supplementary material for: Effect of Driving Pressure-Oriented Ventilation on Patients Undergoing One-Lung Ventilation During Thoracic Surgery: A Systematic Review and Meta-Analysis
Source: Front Surg. 2022 May 27;9:914984. doi: 10.3389/fsurg.2022.914984 (PMC9198650; doi:10.3389/fsurg.2022.914984)
Supplement: Supplementary file 2 [file Table_3_v1.docx]

Supplementary Table 3: Explanation of the driving pressure-oriented group

| Reference | the reason for driving pressure-oriented group |
| --- | --- |
| Park et al  （2019） | The experimental group: Used the PEEP incremental method to titrate PEEP to obtain the lowest driving pressure for driving pressure-directed ventilation.  The control group: Received protective ventilation (PEEP=5cmH2O, V_T_=6ml/kg IBW combined with a certain level of lung recruitment maneuvers). |
| Spadaro et al  （2018） | The experimental group: PEEP=10cmH_2_O, V_T_: 4-5ml/kg PBW  The control group: PEEP=0cmH_2_O, VT: 4-5ml/kg PBW  When PEEP=10cmH_2_O, the driving pressure is 12±3cmH2O;  When PEEP=0cmH_2_O, the driving pressure is 16±3cmH2O(P<0.001)  Therefore, the PEEP=10cmH_2_O group was regarded as the experimental group. That is the driving pressure-oriented group. |
| Liu et al  （2020） | The experimental group: Individualized PEEP by EIT  The control group: PEEP=5cmH_2_O  The driving pressure of the PEEP_EIT_ group was 4.9 cmH_2_O lower than that of the control group (95%CI: 3.8-6.1 cmH2O; P<0.001)  Therefore,we regarded the PEEP_EIT_ group as the driving pressure-oriented group. |
| Rauseo et al  （2018） | The experimental group: Implemented an open lung approach (OLA) strategy (lung recruitment immediately after the optimal static compliance of the respiratory system by titrating PEEP during one-lung ventilation)  Compared with before OLA, driving pressure in the OLV_Post-OLA_ group decreased from 9.2±0.4cmH2O to 6.8±0.6cmH2O;P=0.001 ).  Therefore, we regard the OLV_Post-OLA_ group as the driving pressure guide group |
| Spadaro et al  （2020） | The experimental group: PEEP decrement method and titrated PEEP to the lowest driving pressure from PEEP=16cmH_2_O after lung recruiting maneuvers.  The control group: PEEP=0cmH_2_O  Driving pressure: the experimental group with the control group: 8[7-11]VS14[13-19]cmH2O, P<0.05  So we regarded the experimental group as the driving pressure-oriented group. |
| Xu et al  （2020） | The experimental group: the PEEP_DYN_ group—titrating PEEP from PEEP=4cmH2O until reaching the best dynamic compliance  The control group: PEEP=0cmH_2_O  The PEEP_DYN_ group showed lower driving pressure compared with PEEP=0cmH2O (P<0.05), so we regarded the PEEP_DYN_ group as the driving pressure-oriented group |
| Zhang et al  （2020） | the experimental group: started from PEEP=15cmH2O, and PEEP was titrated down to reach the maximum lung compliance.  the control group: PEEP=5cmH_2_O  Compared with the control group, the individualized PEEP group had lower driving pressure (11.7±3.3 VS 14.8±2.4, P<0.01).  Therefore, we regarded the individualized PEEP group as the driving pressure-oriented group. |
