# Supplementary material for: Effect of Driving Pressure-Oriented Ventilation on Patients Undergoing One-Lung Ventilation During Thoracic Surgery: A Systematic Review and Meta-Analysis
Source: Front Surg. 2022 May 27;9:914984. doi: 10.3389/fsurg.2022.914984 (PMC9198650; doi:10.3389/fsurg.2022.914984)
Supplement: Supplementary file 4 [file Table_5_v1.docx]

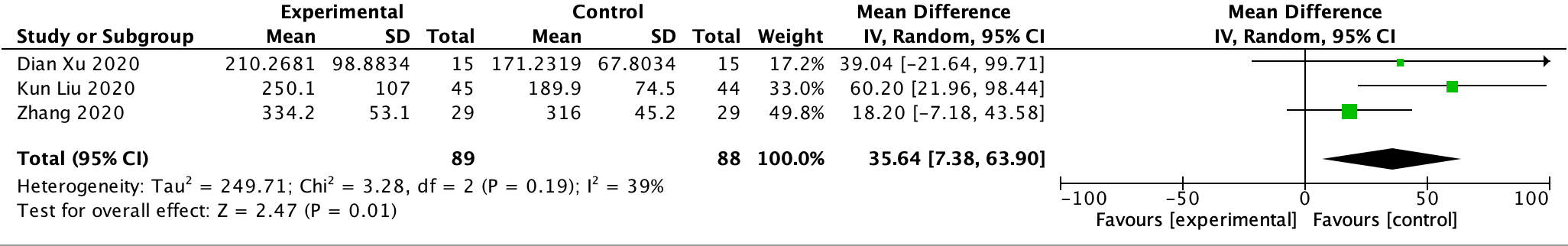


Figure 7: Forest plot of the PaO_2_/FiO_2_ ratio during at 0.5h of one-lung ventilation


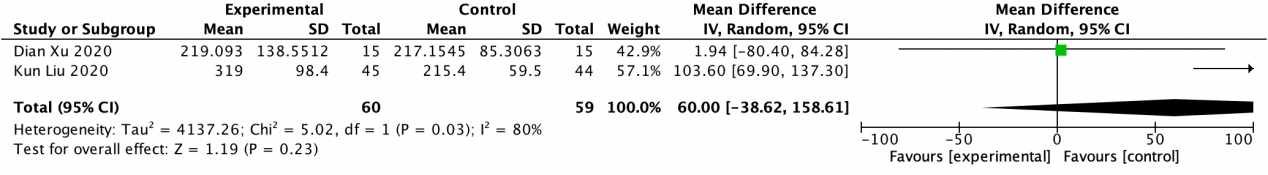


Figure 8: Forest plot of the PaO_2_/FiO_2_ ratio during at 1h of one-lung ventilation


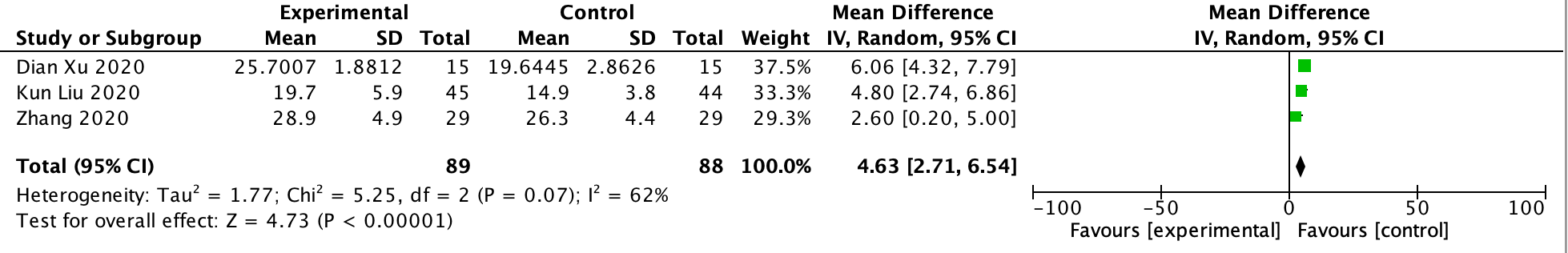


Figure 9: Forest plot of respiratory system compliance at 0.5h during one-lung ventilation.


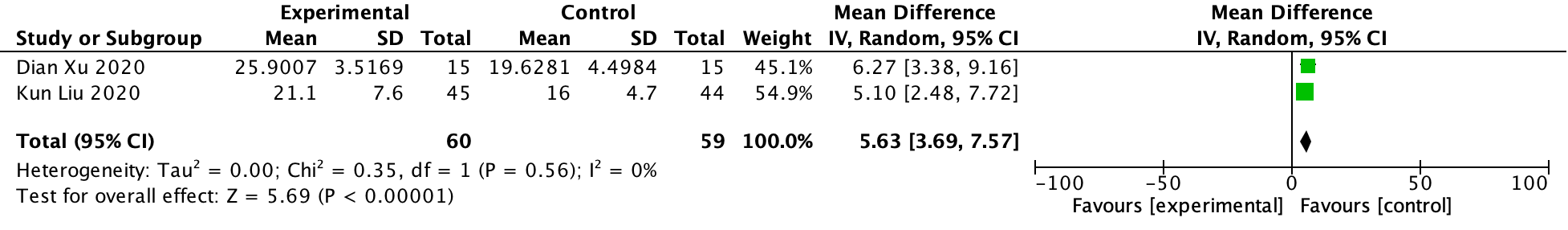


Figure 10: Forest plot of respiratory system compliance at 1h during one-lung ventilation.


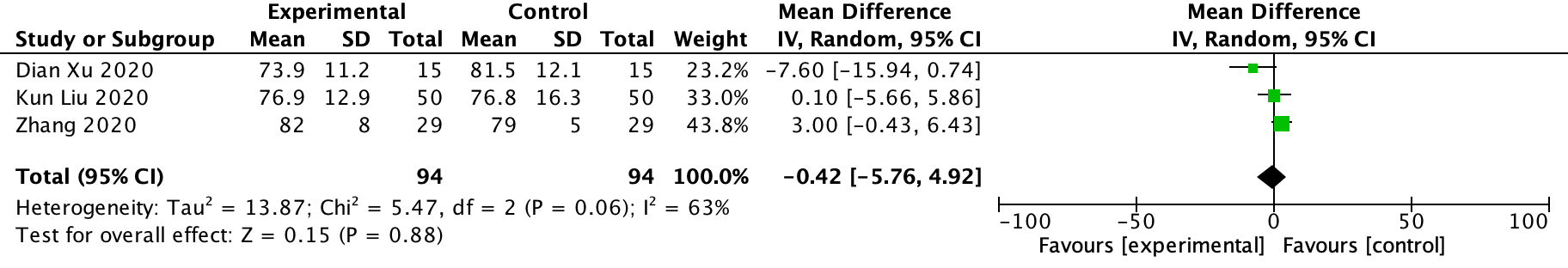


Figure 11: Forest plot of mean arterial pressure at 0.5h during one-lung ventilation


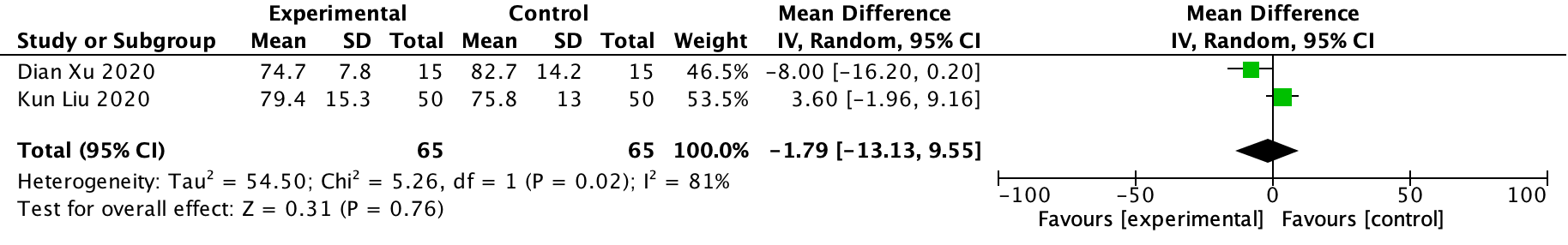


Figure 12: Forest plot of mean arterial pressure at 1h during one-lung ventilation
